# A comparison of various insulin resistance indices and the possibility of hypertension in military adults: CHIEF study

**DOI:** 10.1186/s13098-024-01323-x

**Published:** 2024-04-02

**Authors:** Wei-Che Huang, Kun-Zhe Tsai, Kai-Ti Yang, Han-Hsing Chen, Younghoon Kwon, Gen-Min Lin

**Affiliations:** 1 Department of Internal Medicine, Hualien-Armed Forces General Hospital, No. 100, Jinfeng St., 970 Hualien City, Taiwan; 2 Department of Surgery, Tri-Service General Hospital, National Defense Medical Center, Taipei, Taiwan; 3 Department of Stomatology of Periodontology, Mackay Memorial Hospital, Taipei, Taiwan; 4 Department of Periodontology, School of Dentistry, National Defense Medical Center, Tri-Service General Hospital, Taipei, Taiwan; 5 Department of Surgery, Mennonite Christian Hospital, Hualien, Taiwan; 6 Department of Internal Medicine, Mennonite Christian Hospital, Hualien, Taiwan; 7 Department of Medicine, University of Washington, Seattle, WA USA; 8 Departments of Medicine, Tri-Service General Hospital, National Defense Medical Center, Taipei, Taiwan

**Keywords:** Cohort study, Hypertension, Insulin resistance indices, Young adults

## Abstract

**Background:**

Insulin resistance is associated with the development of hypertension, whereas there were rare studies comparing various non-insulin based insulin resistance (NI-IR) indices for the possibility of hypertension among young and middle-aged adults.

**Methods:**

This cross-sectional study included a total of 4,080 military personnel, aged 18–50 years, without antihypertensive medications therapy in 2014. All subjects received annual health examinations for blood pressure (BP) measurements. Stage I isolated diastolic hypertension (IDH) and isolated systolic hypertension (ISH) and combined hypertension were respectively defined as systolic BP (SBP) < 130 mmHg/diastolic BP (DBP) 80–89 mmHg, SBP 130–139 mmHg/DBP < 80 mmHg, and SBP 130–139 mmHg/DBP 80–89 mmHg. The cut-off values of stage II hypertension for SBP and DBP were 140–159 mmHg and 90–99 mmHg, respectively. Four NI-IR indices included the serum triglycerides (TG) to high-density lipoprotein cholesterol (HDL-C) ratio, TyG index, Metabolic Score for IR (METS-IR) and ZJU index which were defined according to their specific formula. Multiple logistic regression analysis with adjustments for age, sex, anthropometrics, substance use, kidney function, serum uric acid, atherogenic cholesterols and physical activity was performed to determine the associations.

**Results:**

There were 1,024 subjects with hypertension (25.1%) in which 739 were stage I hypertension, and 285 were stage II hypertension. For total hypertension, there were an association with TyG and METS-IR indices [odds ratios (ORs) and 95% confidence intervals: 1.432 (1.215–1.688) and 1.553 (1.040–2.321), respectively]. For hypertension subtypes, TyG index was positively associated with overall, stage I, and stage II ISH [ORs: 1.447 (1.149–1.823), 1.317 (1.029–1.687), and 2.011 (1.351–2.994), respectively], while TG/HDL-C, METS-IR and ZJU indices were merely associated with stage II ISH [ORs: 1.053 (1.006–1.103), 3.001 (1.171–7.696) and 1.009 (1.000-1.017), respectively]. In addition, TyG and METS-IR indices were positively associated with stage II IDH [ORs: 1.813 (1.207–2.721) and 2.85 (1.080–7.520), respectively], and TyG index was also associated with combined hypertension [OR: 1.425 (1.007–1.833)].

**Conclusion:**

Among young and middle-aged adults, insulin resistance assessed by the four NI-IR indices was positively associated with stage II ISH, while only TyG index had a significant association for both stage II IDH and combined hypertension.

## Introduction

Hypertension is widely known as a potent risk factor of cardiovascular diseases (CVD) and cognitive impairment [1, 2], and currently is the leading attributable etiology for the overall mortality in the world, with 10.8 million deaths in 2019 [3]. It is notable that the overall age-standardized rates of hypertension-related CVD mortality increased significantly over the last 5 years [4]. In prior studies, poor awareness of existence of hypertension and poor control of blood pressure (BP) in approximately half of the hypertensive population could account for an increased prevalence of CVD and other comorbidities related to hypertension [5]. As the incidence of hypertension increases abruptly after middle age, it is crucial to clarify early modifiable risk factors of hypertension among young- and middle-aged individuals.

Insulin resistance (IR) characterizes reduced sensitivity and responsiveness to insulin in muscle, liver and β cells, and is linked to impaired glucose tolerance as pre-diabetes [6]. In addition, IR could promote atheroma plaque formation, left ventricular hypertrophy and diastolic dysfunction, subsequently leading to heart failure and CVD [7, 8]. As was known, those with IR had a greater risk of hypertension, and an early acknowledgement of hyperinsulinemia which is the gold-standard biomarker of IR, to identify individuals at high hypertensive risk could be a crucial measure for the primary prevention of the following CVD [9]. Although IR serves as a possible tool to assess the potential risk of hypertension, serum insulin levels are largely affected by the fasting status of individuals [10] and thus not widely applied to the routine health examinations. In the past few decades, some non-insulin based IR (NI-IR) indices, e.g., ratio of triglycerides to high-density lipoprotein cholesterol (TG/HDL-C), TG glucose (TyG) index, metabolic score for IR (METS-IR) and the Zhejiang University (ZJU) index were proposed to show a good correlation to the Homeostatic Model Assessment for Insulin Resistance (HOMA-IR) and found to be a better indicator of metabolic syndrome than HOMA-IR [11–14]. Several studies compared the performance of various NI-IR indices to correlate to prevalent hypertension among middle- and old-aged individuals, while no studies were for young and middle-aged adults. Therefore, this study aimed to examine the associations of various NI-IR indices with hypertension among young and middle-aged adults.

## Methods

### Study population

This cross-sectional study included 4,080 military personnel aged between 18 and 50 years from the Cardiorespiratory Fitness and Health in Eastern Armed Forces (CHIEF) study in Taiwan during 2014. Participants were free of diabetes mellitus and were not using antihypertensive and lipid-lowering medications [15–18]. The CHIEF study was prospectively designed to explore the correlates and associations between physical fitness, potential risk factors and subsequent cardiometabolic comorbidities among physically active military young adults. This study was performed based on the latest Declaration of Helsinki guideline. This study obtained approval from the Institutional Review Board (IRB) of the Mennonite Christian Hospital in Hualien City, Taiwan (No. 16-05-008). Written informed consent was obtained from all individuals, ensuring their voluntary agreement to be part of the research before participating in the study.

### The 2014 military health examinations

During the initial assessment, participants provided information about their substance use status, e.g., alcohol consumption and tobacco smoking (active versus former/never) and moderate coffee intake. In addition, participants also self-reported their levels of moderate-intensity physical activity (PA). The PA levels were classified based on cumulative leisure-time running time per week into < 150 min, 150–299 min and ≥ 300 min over the preceding six months. The collection of this information relied on self-reported responses to a questionnaire administered at the Hualien Armed Forces General Hospital [19–21].

Anthropometric parameters such as waist circumference (WC), body height, and weight of each participant were measured in a standing position. The body mass index (BMI) was computed as the ratio of the participant’s body weight in kilograms to the square of their body height in square meters.

Fasting blood samples were collected after a 12-hour overnight fast from each participant and were utilized to determine serum concentrations of serum uric acid (SUA), blood urea nitrogen (BUN), creatinine, alanine aminotransferase (ALT), aspartate aminotransferase (AST), fasting glucose (FG) and lipid profiles including total cholesterol, low-density lipoprotein cholesterol (LDL-C), HDL-C and TG. These metabolic biomarkers were analyzed utilizing an automated analyzer (Olympus AU640, Kobe, Japan) [22]. Estimated glomerular filtration rate (eGFR) was calculated utilizing the Modification of Diet in Renal Disease (MDRD) formula [23].

### Insulin resistance index calculation

In this study, four NI-IR indices for incident hypertension were compared. The formula of TyG index, was calculated as ln[TG (mg/dL) ×FG (mg/dL)/2] [11]. The TG/HDL-C ratio, was defined by TG (mg/dL) divided by HDL-C (mg/dL) [13]. The METS-IR formula was defined as ln[(2 × FG (mg/dL) + total cholesterol (mg/dL)] ×BMI/ ln[HDL-C (mg/dL)] [12]. The ZJU index was defined as BMI + FG (mmol/L) + TG (mmol/L) + 3 ×ALT (U/L)/AST (U/L) (+ 2, if female) [14].

### Definition of hypertension and phenotypes

Blood pressure (BP) measurements were conducted for participants in a seated position using an oscillometric method through an automatic BP device (FT201 Parama-Tech Co., Ltd, Fukuoka, Japan) [24–28]. If the initial systolic/diastolic BP level exceeded 130/80 mmHg, a second measurement was taken after a 15-minute rest, and the final reported BP level was determined as the average of both measurements [26, 28].

Based on the latest U.S. guidelines [29], hypertension was defined as systolic BP (SBP) ≥ 130 mmHg and/or diastolic BP (DBP) ≥ 80 mmHg. Isolated systolic hypertension (ISH), and isolated diastolic hypertension (IDH), and combined hypertension (CH) were respectively defined as SBP ≥ 130 mmHg/DBP < 80 mmHg, and SBP < 130 mmHg/DBP ≥ 80 mmHg, and SBP ≥ 130 mmHg/DBP ≥ 80 mmHg. The cut-off levels of SBP and DBP for stage I hypertension were 130–139 mmHg and DBP 80–89 mmHg, respectively, while the cut-off levels of SBP and DBP for stage II hypertension were 140–159 mmHg and 90–99 mmHg, respectively. For those developing hypertension, lifestyle modifications were recommended as the initial management, and antihypertensive medications were recommended as an adjuvant therapy for those with stage II hypertension.

### Statistical analysis

Participants were divided into those with hypertension and those without. The clinical characteristics of the study participants were presented as mean and standard deviation for continuous variables and as numbers and percentages for categorical variables. Analysis of variance (ANOVA) and chi-square tests were used to determine the statistical differences in continuous and categorical variables respectively.

Multivariable logistic regression analysis was utilized to examine the odds ratio (OR) and 95% confidence interval (CI) of each NI-IR index (each 1-unit increase) with the possibilities of stage I, stage II and total hypertension. In Model 1, sex, age, tobacco smoking status, alcohol intake status, BMI, WC and PA levels were controlled for. In Model 2, total cholesterol, LDL-C, SUA, BUN and eGFR were further controlled for. The possibilities of stage I and stage II ISH, IDH and CH for each NI-IR index were respectively assessed.

The diagnostic performance of each NI-IR index to determine the presence of hypertension was assessed using the area under the curves (AUC) of receiver operating characteristic (ROC). The sensitivity, specificity and optimal cut-off value of the four NI-IR indices for the presence of hypertension were calculated through ROC analysis. A p-value < 0.05 was regarded as statistical significance. All statistical analyses were performed using SPSS version 26.0 for Windows, developed by IBM Corp. in Armonk, NY, USA.

## Results

### Clinical characteristics of the study population

Of the overall 4,080 study participants, 1,024 subjects had hypertension with an average age of 30.28 ± 6.14 years, SBP of 132.71 ± 9.26 mmHg and DBP of 80.79 ± 10.02 mmHg, and a predominant proportion of men [*N* = 994 (97.1%)], which were found higher than that in 3,056 subjects without hypertension. In addition, all of the NI-IR indices, metabolic biomarkers, kidney functions and PA levels were found greater in those having hypertension. Table 1 reveals further details about the characteristics of participants.

**Table 1:** Clinical characteristics of the military young and middle-aged population

	Those without hypertension (*N* = 3,056)	Those with hypertension (*N* = 1,024)	p-value
Insulin resistance index			
TyG index	8.30 ± 0.55	8.56 ± 0.61	< 0.001
TG/HDL-C	2.31 ± 2.67	3.22 ± 3.89	< 0.001
METS-IR	2.00 ± 0.21	2.10 ± 0.23	< 0.001
ZJU index	125.42 ± 16.08	131.67 ± 17.46	< 0.001
Stage I hypertension			
Isolated systolic hypertension, %	0	364 (35.5)	
Isolated diastolic hypertension, %	0	212 (20.7)	
Combined hypertension, %	0	163 (15.9)	
Stage II hypertension	0	285 (27.8)	
Isolated systolic hypertension, %	0	113 (11.0)	
Isolated diastolic hypertension, %	0	109 (10.6)	
Combined hypertension, %	0	63 (6.2)	
Age, years	28.16 ± 5.81	30.28 ± 6.14	< 0.001
Male sex, %	2675 (87.5)	994 (97.1)	< 0.001
Active alcohol intake, %	1207 (39.5)	479 (46.8)	< 0.001
Active tobacco smoking, %	1069 (35.5)	357 (34.9)	0.90
Daily coffee intake ≥ 4 cups or 600 mL, %			
Moderate-intensity PA levels, %			
<150 min/wk	697 (22.8)	198 (19.3)	0.03
150–299 min/wk	1165 (38.1)	389 (38.0)	
≥300 min/wk	1194 (39.1)	437 (42.7)	
SBP, mmHg	111.96 ± 10.33	132.71 ± 9.26	< 0.001
DBP, mmHg	66.41 ± 7.19	80.79 ± 10.02	< 0.001
Waist circumference, cm	81.20 ± 8.30	85.88 ± 7.94	< 0.001
Body mass index, kg/m^2^	24.17 ± 3.07	26.00 ± 2.99	< 0.001
Blood tests			
Total cholesterol, mg/dL	171.56 ± 32.85	180.06 ± 35.17	< 0.001
LDL-C, mg/dL	103.12 ± 29.10	109.42 ± 30.19	< 0.001
HDL-C, mg/dL	49.19 ± 10.28	47.51 ± 10.17	< 0.001
TG, mg/dL	102.56 ± 81.60	137.39 ± 128.01	< 0.001
FPG, mg/dL	92.66 ± 12.68	94.62 ± 13.94	< 0.001
SUA, mg/dL	6.40 ± 1.37	6.91 ± 1.42	< 0.001
BUN, mg/dL	12.62 ± 2.85	12.87 ± 2.93	0.02
eGFR, mL/min/1.73m^2^	101.40 ± 14.93	98.66 ± 14.73	< 0.001

Continuous variables are expressed as mean ± SD (standard deviation), and categorical variables as N (%)

*Abbreviations* TyG, triglyceride glucose; TG, triglycerides; HDL-C, high-density lipoprotein cholesterol; METS-IR, metabolic score for insulin resistance; ZJU, Zhejiang University; PA, physical activity; SBP, systolic blood pressure; DBP, diastolic blood pressure; PP, pause pressure; LDL-C, low-density lipoprotein cholesterol; FPG, fasting plasma glucose; SUA, serum uric acid; BUN, blood urea nitrogen; eGFR, estimated glomerular filtration rate

### NI-IR indices and the possibilities of hypertension

Table 2 reveals the multivariable adjusted-associations of various NI-IR indices with stage I, stage II and total hypertension. All of the TyG, TG/HDL-C, METS-IR, and ZJU indices were associated with a greater possibility of hypertension of overall and each stage in crude Model, while all of the associations remaining significant in Model 1 and Model 2 were only observed for the TyG index [ORs and 95% CIs for total hypertension: 1.448 (1.256–1.670) and 1.432 (1.215–1.688), respectively; for stage I hypertension: 1.286 (1.096–1.510) and 1.292 (1.074–1.554), respectively; and for stage II hypertension: 1.907 (1.530–2.375) and 1.834 (1.403–2.397), respectively]. With regard to the TG/HDL-C, METS-IR and ZJU indices, all of the possibilities for stage I hypertension were not significant in Models 1 and 2. However, all of the possibilities for stage II hypertension were significant in Model 1 (ORs: 1.062 (1.027–1.097), 3.241 (1.752–5.994) and 1.008 (1.002–1.014), respectively), while only the METS-IR was associated with a greater possibility with stage II hypertension [OR: 2.415 (1.275–4.573)].

**Table 2:** Associations of various insulin resistance indices with overall, stage I and stage II hypertension

Total hypertension						
	Crude model		Model 1		Model 2	
	OR (95% CI)	p-value	OR (95% CI)	p-value	OR (95% CI)	p-value
TyG index	2.181 (1.924–2.472)	< 0.001	1.448 (1.256–1.670)	< 0.001	1.432 (1.215–1.688)	< 0.001
TG/HDL-C	1.121 (1.088–1.155)	< 0.001	1.035 (1.008–1.062)	0.01	1.024 (0.995–1.053)	0.10
METS-IR	6.778 (4.917–9.343)	< 0.001	1.769 (1.195–2.619)	0.004	1.553 (1.040–2.321)	0.03
ZJU index	1.023 (1.018–1.029)	< 0.001	1.004 (0.999–1.009)	0.08	1.003 (0.998–1.008)	0.27
**Stage I hypertension**						
TyG index	1.900 (1.654–2.182)	< 0.001	1.286 (1.096–1.510)	0.002	1.292 (1.074–1.554)	0.007
TG/HDL-C	1.010 (1.066–1.136)	< 0.001	1.015 (0.983–1.049)	0.35	1.007 (0.971–1.045)	0.70
METS-IR	5.143 (3.613–7.323)	< 0.001	1.360 (0.873–2.119)	0.17	1.246 (0.789–1.968)	0.34
ZJU index	1.022 (1.016–1.027)	< 0.001	1.002 (0.996–1.007)	0.58	1.001 (0.995–1.007)	0.72
**Stage II hypertension**						
TyG index	3.015 (2.492–3.647)	< 0.001	1.907 (1.530–2.375)	< 0.001	1.834 (1.403–2.397)	< 0.001
TG/HDL-C	1.153 (1.113–1.195)	< 0.001	1.062 (1.027–1.097)	< 0.001	1.038 (1.000–1.078)	0.053
METS-IR	13.159 (8.053–21.502)	< 0.001	3.241 (1.752–5.994)	< 0.001	2.415 (1.275–4.573)	0.007
ZJU index	1.030 (1.023–1.036)	< 0.001	1.008 (1.002–1.014)	0.008	1.005 (0.998–1.011)	0.15

Data are presented as odds ratio (OR) and 95% confidence intervals (CI) using multiple logistic regression analysis for the association between each insulin resistance index (each 1-unit increase) and prevalent hypertension

Model 1: age, sex, alcohol intake, tobacco smoking, physical activity level, waist circumference, and body mass index adjustments

Model 2: Model 1 covariates + total cholesterol, low-density lipoprotein cholesterol, serum uric acid, blood urea nitrogen and estimated glomerular filtration rate adjustments

*Abbreviations* TyG, triglyceride glucose; TG, triglycerides; HDL-C, high-density lipoprotein cholesterol; METS-IR, metabolic score for insulin resistance; ZJU, Zhejiang University

### NI-IR indices and the possibilities of subtype hypertension

Table 3 reveals the results of the multivariable-adjusted associations of various NI-IR indices with overall, stage I, and stage II subtype hypertension including ISH, IDH and CH. The TyG index was significantly associated with a greater possibility of overall, stage I and stage II ISH (ORs: 1.447 (1.149–1.823), 1.317 (1.029–1.687) and 2.011 (1.351–2.994, respectively), overall and stage II IDH (ORs: 1.408 (1.061–1.869) and 1.813 (1.207–2.721), respectively), and overall CH [OR: 1.425 (1.107–1.833)]. The TG/HDL-C ratio was significantly associated with a greater possibility of stage II ISH [OR: 1.053 (1.006–1.103)]. The METS-IR index was significantly associated with a greater possibility of stage II ISH and IDH (ORs: 3.001 (1.171–7.696) and 2.850 (1.080–7.520, respectively). Finally, the ZJU index was associated with a greater possibility of overall and stage II ISH (ORs: (ORs: 1.007 (1.001–1.013) and 1.009 (1.000-1.017, respectively).

**Table 3:** Associations of various insulin resistance indices with overall, stage I and stage II hypertension subtypes

Total hypertension						
	Isolated systolic hypertension		Isolated diastolic hypertension		Combined hypertension	
	OR (95% CI)	p-value	OR (95% CI)	p-value	OR (95% CI)	p-value
TyG index	1.447 (1.149–1.823)	0.002	1.408 (1.061–1.869)	0.01	1.425 (1.107–1.833)	0.006
TG/HDL-C	1.025 (0.986–1.066)	0.21	1.017 (0.975–1.061)	0.43	1.021 (0.986–1.058)	0.24
METS-IR	1.475 (0.833–2.610)	0.18	1.510 (0.759–3.005)	0.24	1.565 (0.856–2.863)	0.14
ZJU index	1.007 (1.001–1.013)	0.03	1.002 (0.995–1.010)	0.54	0.997 (0.989–1.005)	0.99
**Stage I hypertension**						
TyG index	1.317 (1.029–1.687)	0.02	1.218 (0.890–1.665)	0.21	1.337 (0.936–1.911)	0.11
TG/HDL-C	1.001 (0.949–1.055)	0.98	1.008 (0.953–1.065)	0.78	1.014 (0.952–1.080)	0.66
METS-IR	1.157 (0.624–2.147)	0.64	1.081 (0.502–2.328)	0.84	1.677 (0.711–3.953)	0.23
ZJU index	1.004 (0.997–1.012)	0.26	1.002 (0.992–1.011)	0.73	0.990 (0.975–1.005)	0.18
**Stage II hypertension**						
TyG index	2.011 (1.351–2.994)	0.001	1.813 (1.207–2.721)	0.004	1.645 (0.956–2.830)	0.07
TG/HDL-C	1.053 (1.006–1.103)	0.02	1.034 (0.984–1.085)	0.18	1.023 (0.960–1.091)	0.48
METS-IR	3.001 (1.171–7.696)	0.02	2.850 (1.080–7.520)	0.03	1.258 (0.337–4.698)	0.73
ZJU index	1.009 (1.000–1.017)	0.03	1.003 (0.992–1.013)	0.62	1.001 (0.988–1.015)	0.86

Data are presented as odds ratios (OR) and 95% confidence intervals (CI) using multivariable logistic regression analysis for the association between each insulin resistance index (each 1-unit increase) and prevalent hypertension with adjustments for age, sex, alcohol intake, tobacco smoking, physical activity level, waist circumference, body mass index, total cholesterol, low-density lipoprotein cholesterol, serum uric acid, blood urea nitrogen and estimated glomerular filtration

*Abbreviations* TyG, triglyceride glucose; TG, triglycerides; HDL-C, high-density lipoprotein cholesterol; METS-IR, metabolic score for insulin resistance; ZJU, Zhejiang University

### Diagnostic performance of NI-IR indices

Table 4 reveals the ROC curve analysis for the presence of overall and stage II hypertension. The greatest AUCs under the ROC curve for total hypertension is 0.634 (0.614–0.654) with the ZJU index, followed by 0.633 (0.613–0.653) with the TyG index, 0.628 (0.608–0.648) with the METS-IR index and the lowest one of 0.623 (0.603–0.643) with the TG/HDL-C ratio. In contrast, the highest AUCs under the ROC curve for the presence of stage II hypertension is 0.661 (0.626–0.695) with the TyG index, followed by 0.650 (0.615–0.685) with the ZJU index, 0.646 (0.612–0.681) with the TG/HDL-C ratio, and the lowest one of 0.642 (0.609–0.676) with the METS-IR. In addition, the cut-off levels for each index to achieve the optimal outcomes, as evaluated by sensitivity and specificity, in predicting the likelihood of hypertension is also shown in Table 4.

**Table 4:** Receiver Operating Characteristic (ROC) curve analysis for the presence of total and stage II hypertension

Total hypertension						
	Cut-off point	Sensitivity	Specificity	AUC	95% CI	p-value
TyG index	8.454	0.548	0.660	0.633	0.613–0.653	< 0.001
TG/HDL-C	2.139	0.554	0.645	0.623	0.603–0.643	< 0.001
METS-IR	2.008	0.644	0.560	0.628	0.608–0.648	< 0.001
ZJU index	127.130	0.573	0.629	0.634	0.614–0.654	< 0.001
**Stage II hypertension**						
TyG index	8.359	0.690	0.563	0.661	0.626–0.695	< 0.001
TG/HDL-C	2.172	0.621	0.621	0.646	0.612–0.681	< 0.001
METS-IR	2.008	0.697	0.525	0.642	0.609–0.676	< 0.001
ZJU index	131.245	0.513	0.719	0.650	0.615–0.685	< 0.001

*Abbreviations* TyG, triglyceride glucose; TG, triglycerides; HDL-C, high-density lipoprotein cholesterol; METS-IR, metabolic score for insulin resistance; ZJU, Zhejiang University; AUC, area under curve; CI, confidence interval

## Discussion

The principal findings of this study were that among young adults, all of the four NI-IR indices were positively associated with a higher possibility of stage II ISH. Both TyG and METS-IR indices were positively associated with a higher possibility of overall hypertension and stage II hypertension, particularly IDH. In addition, only TyG index was positively associated with a greater possibility of CH. With regard to the integrated performance, the TyG and ZJU indices were noted with the greatest capacities to identify the presence of overall and stage II hypertension.

Recognizing IR among individuals with hypertension is integral for effectively managing hypertension, not only in the Western but also in the Asian Countries. Many cross-sectional studies have confirmed an association between the TyG index and the possibility of hypertension in middle- and old-aged individuals [10, 11]. Our findings were consistent with the prior studies, reinforcing the link between increased TyG index and hypertension among young adults. In one study the TyG index and the METS-IR index performed similarly in identifying metabolic syndrome [30]. Similarly, for the prediction capacity of the presence of hypertension, the TyG index was found greater than the METS-IR index in the present study. It was notable that another study demonstrated that for a significant association between the METS-IR index and prevalent hypertension particularly in normal-weight Chinese adults [12].

There have been few cross-sectional studies to examine the association between the TG/HDL-C ratio and prevalent hypertension. One such study, involving 112,798 participants in China, revealed an association between the TG/HDL-C ratio and prevalent hypertension while defined as resting BP ≥ 140/90 mmHg [13]. However, another study showed no significant association between the TG/HDL-C ratio and prevalent hypertension as defined as resting BP ≥ 130/85 mmHg [12]. In the present study, the association of the TG/HDL-C ratio with prevalent hypertension as defined as resting BP ≥ 130/80 mmHg was no longer statistically significant after further adjustments for total cholesterol, LDL-C, SUA and kidney function, whereas the association was borderline significant if hypertension was defined as BP ≥ 140/90 mmHg, consistent with the findings in prior studies [12, 13].

The ZJU index was initially proposed to predict the presence of non-alcoholic fatty liver disease (NAFLD) in a Chinese population, which has been regarded as a metabolic disorder of liver [14]. The index incorporated BMI and standard laboratory tests e.g., metabolic biomarkers other than the metabolic syndrome components and liver function [14]. NAFLD is characterized by the excessive accumulation of fat in hepatocytes without discernible alternative causes, and it is associated with a greater risk of CVD [31]. Although there were no studies to investigate the association between the ZJU index and hypertension, the ZJU index as other NI-IR indices was likely related to hypertension in this study. IR stands as a fundamental pathological characteristic of metabolic syndrome and is recognized as a risk factor for the onset of hypertension due to an increased sympathetic nervous system activity [32]. In addition, IR can increase BP by enhancing the synthesis and release of endothelin and activating the renin-angiotensin-aldosterone system [33]. Both the mechanisms can lead to arterial stiffness, accounting for the dominant subtype of hypertension, ISH, in this study [32, 33].

### Strengths and limitations

The major strength of the present study is that it is the first report to compare the associations of various NI-IR indices with hypertension in young adults. In addition, this study had a large number of individuals and the associations were adjusted for potential confounders to minimize bias. On the contrary, the main limitation is that causal inference cannot be made due to the nature of the cross-sectional study. The study subjects exclusively consisted of the military personnel in Taiwan, possibly restricting the generalizability and applicability of the findings to the general population of other ethnic young adult groups. Further research is necessary to confirm the applicability of these findings in diverse populations. Finally, since approximately 90% of the population were men, we could not examine the sex-specific associations between each NI-IR index and hypertension.

## Conclusion

Our findings endorse that insulin resistance as assessed by the TyG, TG/HDL-C, METS-IR, and ZJU indices was positively associated with the possibility of hypertension among young and middle-aged adults. It was noted that TyG and MetS indices were associated with stage II ISH and IDH, whereas TG/HDL and ZJU indices were only associated with stage II ISH. Of the four NI-IR indices, the TyG index and the ZJU index may have the highest predictive capacity for the presence of hypertension.

## Data Availability

The datasets generated and/or analyzed during the current study are not publicly available due to materials obtained from the military in Taiwan, which were confidential, but are available from the corresponding author on reasonable request.

## Abbreviations

ALT: Alanine aminotransferase
AST: Aspartate aminotransferase
AUC: Area under curve
BMI: Body mass index
BP: Blood pressure
CH: Combined hypertension
CHIEF: Cardiorespiratory fitness and Health in Eastern armed Forces
CI: Confidence intervals
DBP: Diastolic Blood Pressure
eGFR: Estimate glomerular filtration rate
FG: Fasting plasma glucose
HDL-C: High-Density Lipoprotein Cholesterol
HOMA: Homeostatic Model Assessment
HRs: Hazard Ratios
IDH: Isolated Diastolic Hypertension
ISH: Isolated Systolic Hypertension
IR: Insulin Resistance
IRB: Institutional Review Board
LDL-C: Low-Density Lipoprotein Cholesterol
MDRD: Modification of Diet in Renal Disease
MEST-IR: Metabolic Score for Insulin Resistance
NHANES: National Health And Nutrition Examination Survey
NI-IR: Non-insulin-based Insulin Resistance
PA: Physical Activity
PEACE: Patient-centered Evaluation Assessment of Cardiac Events
ROC: Receiver Operating Characteristic
SBP: Systolic Blood Pressure
SD: Standard Deviation
TG: Triglyceride
TyG: Triglyceride Glucose
ZJU: Zhejiang University
